# Awareness about Vulvovaginal Aesthetics Procedures among Medical Students and Health Professionals in Saudi Arabia

**DOI:** 10.1055/s-0041-1725050

**Published:** 2021-04-15

**Authors:** Shazia Iqbal, Khalid Akkour, Bushra Bano, Ghaiath Hussain, Manal Khalid Kamal Ali Elhelow, Atheer Mansour Al-Mutairi, Balqees Sami Khaza'l Aljasim

**Affiliations:** 1 Alfarabi College of Medicine Riyadh, Alfarabi, Kingdom of Saudi Arabia; 2 King Saud University, Saudi Arabia; 3 Allama Iqbal Medical College, Lahore, Pakistan; 4 Birmingham University, Birmingham, United Kingdom; 5 Department of Clinical Sciences, Alfarabi College of Medicine, Alfarabi, Kingdom of Saudi Arabia

**Keywords:** vulvovaginal cosmetic procedures, vulvovaginal aesthetics procedures, awareness about aesthetic surgery among health professionals, aesthetic gynecology, sexual and reproductive health

## Abstract

**Objective** The present study aimed to explore the opinion and ethical consideration of vulvovaginal aesthetics procedures (VVAPs) among health professionals and medical students in Saudi Arabia.

**Methods** This is a cross-sectional study performed between January 2020 and April 2020. Data was collected through electronic media, WhatsApp, and emails. The results were analyzed by applying the Students t-test, and correlations were considered significant if they presented a p-value < 0.05.

**Results** There is significant demand to educate doctors, health professionals, medical students, and gynecologists for the VVAPs to have a solid foundation, justified indications, and knowledge about various aesthetic options. Although female doctors, medical students, young doctors, and gynecologists have more knowledge about VVAPs, all health professionals ought to be aware of recent trends in vulvovaginal aesthetics (VVA). The present analysis determined that VVA should be under the domain of gynecologists, rather than under that of plastic surgeons, general surgeons, and cosmetologists. The majority of the participants considered that vaginal rejuvenation, “G-spot” augmentation, clitoral surgery, and hymenoplasty are not justifiable on medical grounds.

**Conclusion** The decision to opt for different techniques for vaginal tightening and revitalization should be taken very carefully, utilizing the shared decision-making approach. Ethical aspects and moral considerations are important key factors before embarking in the VVAPs purely for cosmetic reasons. Further research is required to determine the sexual, psychological, and body image outcomes for women who underwent elective VVAPs. Moreover, medical educators must consider VVAPs as part of the undergraduate and postgraduate medical curriculum.

## Introduction

Presently, vulvovaginal aesthetics procedures (VVAPs) are being marketed and promoted by surgeons, while the evidence of their safety and efficacy is still questionable. There are serious concerns regarding medical ethics, morals, and principles about aesthetic procedures in gynecology. Evidence-based recommendations for these aesthetic procedures are scarce. There are no clear guidelines nor convincing pieces of evidence to consider the implications of this practice. There are only a few studies that addressed the concerns of perimenopausal women regarding reproductive health and sexual health. 1 2 Additionally, there is a lack of research that mentions the concerns of women in postoperative vulvovaginal oncology cases to preserve the quality of sexual well-being. 3

Recently, there is a dramatic change in trends of genital aesthetics in a different part of the word. 4 In a few countries, the use of laser treatment for vaginal rejuvenation and tightening is trendy and effective. 5 A variety of procedures has emerged, including reduction labiaplasty, hymenoplasty, G-spot augmentation, and vaginal laxity. Moreover, many nonsurgical procedures are being promoted as simple, safe, and as improving sexual pleasure worldwide. In the middle east, little is researched regarding awareness of vulvovaginal aesthetic (VVA) procedures among health professionals. Moreover, time demands to examine the indications and the standardization of training for gynecologists regarding the awareness of different indications and a vast range of VVAPs.

In the perimenopausal age group, genitourinary problems are common in the form of vaginal atrophy, laxity, vulvovaginal prolapse, vulval burning pain, urinary incontinence, etc. These changes lead to low self-esteem and reduce the quality of sexual health among some women. We have observed the various nonsurgical and surgical treatment options in the practice; for example, the use of polycarbophil-based cream in postmenopausal women, vaginal tightening by lasers, and surgical procedures. 6 To treat lichen sclerosis and lichen planus, some studies suggested the use of autologous platelet-rich plasma intradermal injections to treat vulval lichen sclerosis. 7 Various therapeutic options have been adopted by health care providers for treating vaginal atrophy to improve the quality of sexual health; for example, injectable hyaluronic acid plus calcium hydroxyapatite along with different surgical options. 8 9 10

There is a considerable gap in research to assess the readiness of health care professionals before embarking in the aesthetic procedures and in the practice on patients. The present article explored the responsiveness of health professionals and medical students about VVA procedures in Saudi Arabia. The authors will recommend a model for the enhancement of knowledge and awareness about VVAPs among health providers.

There is hardly any data that support the awareness of VVAPs procedures in the Middle East and Saudi Arabia. Regarding ethical concerns related to VVAPs, it is important to inquire in their own culture and Islamic region. 11 Cultural beliefs play a great impact in the adoption of recent trends in aesthetic gynecology and implement evidence-based practice. Along with awareness of the patients, it is equally important to inquire about the perceptions of healthcare professionals and medical students in a geographical region. It will help to analyze the diverse cultural context about familiarity and the impact of VVAPs procedures in a medical and ethical context. The present study aimed to explore the awareness of VVAPs among health professionals and medical students in Saudi Arabia.

## Methods

We conducted a cross-sectional study by designing a comprehensive Google Docs survey, and descriptive analysis was performed. We conducted the present study between January 2020 and April 2020, in Saudi Arabia. The internal reviewer board of the research unit approved the study at the Alfarabi College of Medicine Riyadh. We distributed the survey through electronic media, WhatsApp, and emails. All participants were informed about the aim of the study, and we explained a brief description of the purpose of the study. The participants declared consent for contribution before filling the survey. A descriptive analysis of the results was performed, and we presented statistics in graphs and charts. To determine correlations, the results were analyzed by applying the Students t-test, and we considered correlations as being significant if they presented a p-value < 0.05. The internal reviewer board of the research unit approved the study at Alfarabi College of Medicine Riyadh.

All practicing Saudi medical licensed (SML) medical doctors, Saudi registered health professionals, consultants of all specialties, residents, registrars, and medical students in Saudi Arabia in the public and private sectors. We included medical students of public and private medical schools. Any nonpracticing doctor out of practice for > five years was excluded from the study, considering their lack of knowledge regarding the health updates on reproductive and sexual health. We also excluded paramedical staff and allied health professionals. The authors of the present study prepared a research questionnaire, and a pilot study established the validity of this questionnaire on 70 participants to establish significant findings. We divided the questionnaire into three sections, and each section had an elaborated description mentioning the facts and purpose of the study. The first section regarded demographic details including age, gender, level of qualification, employability status, years of working experiences as a health professional, specialty type, and the category of health sectors (public or private).

The opinions of the participants about any medical justification and ethical objections against VVAPs were mentioned in the second section. Some details and clarifications about the procedures were explained to the participants. The four-point Likert scale was used in the survey. We asked the participants to answer whether several VVAPs were medically justiﬁable, with the following answer options:

- It is not justiﬁable
- It is rarely justiﬁable
- It is justiﬁable
- It is highly justifiable

The authors questioned the participants about the possible benefits of VVAPs, and we assessed general proclamations and guidance on this topic in the third section. Regarding practical issues about the VVAPs (minimum age of performance, whether it should be performed in public hospitals, etc.), an evaluation was made using a four-point Likert scale with the following options:

- Agree
- Partially agree
- Neither agree nor disagree (neutral)
- Disagree

Concerning the sample collection, we collected data from 260 participants among the 350 expected participants. After the third reminder, the response rate was of 74%, which was considered acceptable to establish results.

## Results

Regarding the demographic data, 72.9% were < 25 years old (freshmen residents, medical students). More than half of them (65.7%) had < 5 years of experience working in the health profession. Among the 260 participants of the study, most of them were female (79.6%) and worked in public hospitals (60.31%). Most of them were medical students (45%), followed by gynecology trainees (13.4%), and gynecology consultants (7.6%). Table 1 summarizes the sociodemographic characteristics of the participants.

**Table 1:** Sociodemographic characteristics of 260 participants

Characteristics of participants ( *n* = 260)	Frequency	Percentage
Gender		
Male	53	21%
Female	207	79.6%
Age (years old)		
20–25	165	63.4%
26–40	75	28.8%
41–50	15	5.7%
> 50	5	1.9%
Working in Region		
Central area	161	61.9%
Southern area	65	25.0%
Western area	27	10.3%
Eastern area	4	1.5%
Northern area	3	1.1%
Educational level		
Medical students	117	45%
MBBS Graduates	48	18.4%
Postgraduate trainee gynecology	35	13.4%
Postgraduate trainee surgery	30	11.5%
Consultant gynecology	20	7.6%
Other specialties	10	3.8%
Duration of employment (years)		
1–5	171	65.7%
5–10	69	26.5%
> 10	20	7.6%

**Fig. 1:**
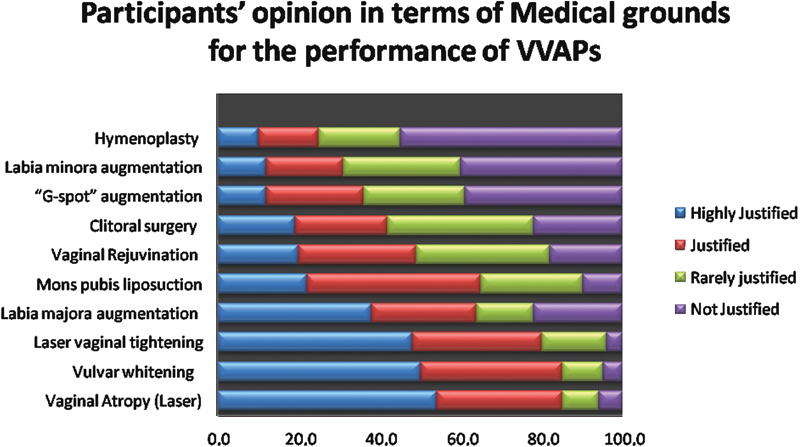
Percentage of participant's opinion about the justification of various vulvovaginal aesthetics procedures on medical grounds ( *n* = 260).

About 68.7% of the participants did not have much knowledge about VVAPs. More than half of the participants agreed that VVAPs on medical grounds is justified for vaginal tightening (by laser), vaginal atrophy (by laser) and whitening of the vulva. However, the majority of the participants considered that vaginal rejuvenation, “G-spot” augmentation, clitoral surgery, and hymenoplasty are not justifiable on medical grounds, as shown in Fig. 1 .

**Fig. 2:**
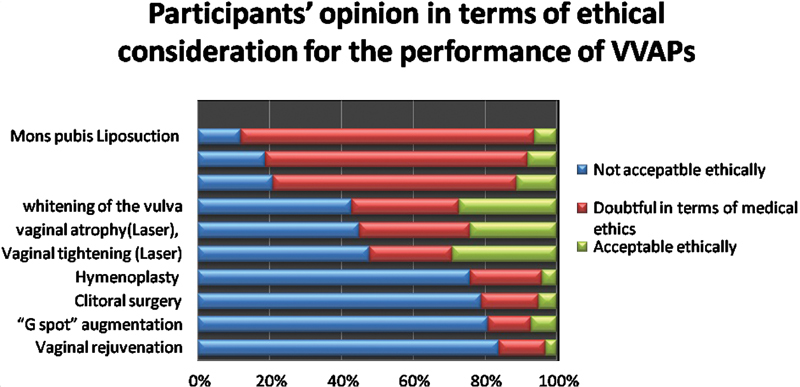
Percentage of participant's opinion about the justification of various vulvovaginal aesthetics procedures on an ethical basis ( *n* = 260).

Regarding ethical considerations, more than half of the participants considered that vaginal rejuvenation, “G-spot” augmentation, clitoral surgery, and hymenoplasty are not ethically accepted, as shown in Fig. 2 . It was determined that aesthetic procedures should be performed by gynecologists rather than by plastic surgeons or general surgeons ( *p* < 0.025), as shown in Table 2 . According to the opinion of the participants, VVAPs related to vaginal tightening and vaginal atrophy are associated with enhanced self-esteem and quality of reproductive/sexual life ( *p* < 0.013), as shown in Table 3 . However, almost all specialty consultants considered that VVAPs are overpriced procedures. However, there was no significant difference in the opinions of private and public health professionals regarding the pricing of different VVAPs ( *p* = 0.05).

**Table 2:** Percentage of participants who considered that vulvovaginal aesthetics procedures should be performed by gynecologists ( *n* = 260)

VVAPs	Private practice			Specialty %			
	Yes	No	*p-value*	ObGyn	Plastic surgery	Other surgical	*p-value*
Hymenoplasty	56.6%	43.1%	0.051	29.6%	8.3%	22.0%	0.025
Augmentation of the labia minora	73.2%	36.9%	0.030	41.3%	12.5%	25.0%	0.021
“G-spot” augmentation	60.1%	39.2%	0.051	49.3%	12.5%	21.3%	0.011
Clitoral surgery	48.3%	41.0%	0.041	44.9%	16.7%	22.9%	0.014
Vaginal rejuvenation	42.8%	14.9%	0.022	54.3%	16.7%	24.1%	0.021
Augmentation of the labia majora	45.3%	41.9%	0.052	51.5%	13.6%	28.4%	0.025
Mons pubis liposuction	42.5%	50.0%	0.059	56.5%	17.4%	37.0%	0.025
Laser Vaginal tightening	52.1%	35.1%	0.041	57.8%	26.1%	21.0%	0.001
Whitening	49.2%	47.3%	0.057	68.0%	30.4%	12.9%	0.001
Vaginal laser (atrophy)	38.5%	60.7%	0.560	65.0%	37.5%	18.1%	0.003

Abbreviation: VVAPs, vulvovaginal aesthetic procedures.

**Table 3:** Percentage of participants' opinion about vulvovaginal aesthetics procedures enhancing the quality of life and correlation between difference of opinion among different health professionals ( *n* = 260)

VVAPs	Considering plastic surgery %	Health professionals opinion about VVAPs enhancing quality of life			
	Yes	Medical students	Residents	Specialists	*p-value*
Hymenoplasty	24.6%	55.8%	34.6%	21.1%	0.048
Augmentation of the labia minora	38,2%	53.8%	31.3%	21.1%	0.001
“G-spot” augmentation	39.1%	37.7%	22.7%	30.6%	0.001
Clitoral surgery	26.3%	45.1%	35.0%	24.2%	0.015
Vaginal rejuvenation	31.4%	45.6%	27.6%	24.2%	0.005
Augmentation of the labia majora	66.1%	56.8%	30.5%	21.4%	0.043
Mons pubis Liposuction	66.5%	60.8%	30.1%	31.8%	0.051
Laser Vaginal tightening	69.5%	62.9%	26.1%	36.0%	0.013
Whitening	74.2%	64.9%	23.7%	17.0%	0.057
Vaginal laser (atrophy)	84.9%	71.8%	20.7%	26.7%	0.013

Abbreviation: VVAPs, vulvovaginal aesthetic procedures.

## Discussion

Currently, women are facing a dilemma regarding freedom of choice related to a long list of VVAPs and female genital cosmetic surgery. Some procedures are indicated on medical grounds but are not ethically accepted in the region. Our research found that there is a significant demand to educate doctors, health professionals, medical students, and gynecologists regarding VVAPs in order to have a solid foundation, justified indications, and knowledge about various aesthetic options. Skilled health professionals should have optimal expertise to test signs and symptoms of the patients and involve a multidisciplinary health team for management. 12 Although medical students, young doctors, and gynecologists have more knowledge about VVAPs, all health professionals must know recent trends in VVA. 13 14

Regarding the ethical aspects of VVAPs, our results are consistent with the Royal College of Obstetrics and Gynecology. 15 This analysis recommends that ethical opinion for patients opting for female genital cosmetic procedures needs to be discussed carefully with microscopic details and should be opted only for indicated patients; for example, if sexual health is compromised physically (atrophy or laxity) and psychologically. Although the health professionals had below-average knowledge about different types of procedures, the majority had a positive attitude toward female genital cosmetic surgery if the concerns of the patient are associated with sexual and psychological satisfaction.

The present study determined that health professionals perceived that VVAPs improve the quality of sexual health and reduce female sexual dysfunctions. 16 Therefore, they would recommend these procedures to indicated patients. Additionally, the results revealed that, in our conservative culture, aesthetic deprivation was observed in general among health providers. 17 Therefore, health providers need to enhance awareness about the variety of procedures and improve their counseling skills to determine the impact of the decision to submit patents to surgery on the psychological and sexual well-being of the patients. 18 Particularly, VVAPs recommendations for uterovaginal prolapse and postoperative oncology cases should be justified to boost the self-esteem of the patients. 19 20

The present analysis determined that VVA ought to be under the domain of gynecologists rather than under those of plastic surgeons and cosmetologists. 21 22 Moreover, the decision to opt for different techniques for vaginal tightening and revitalization should be taken very carefully, utilizing the shared decision-making approach. 15 Ethical aspects and moral considerations are key points to keep in mind before embarking in the VVAPs purely for cosmetic reasons. 23 The decision about VVAPs in menopausal women needs special considerations, and one must give priority to conservative management rather than to surgery. 24 25 There should be evidence-based guidelines and algorithms to be followed by practitioners to help in decisions regarding VVAPs. 14 26 27

**Fig. 3:**
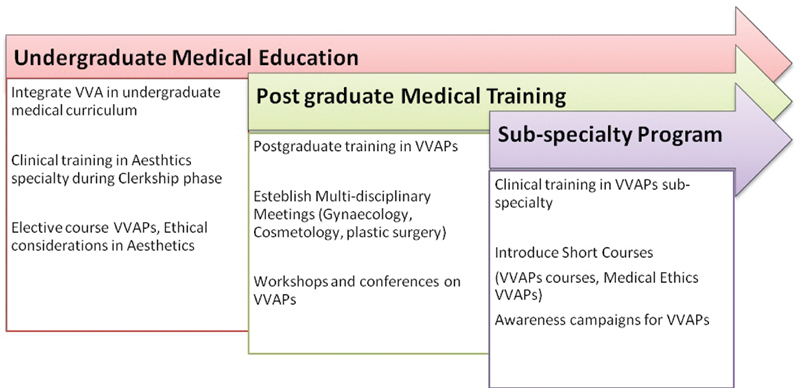
Step by step integration of awareness and knowledge about vulvovaginal aesthetics procedures in medical education.

The authors have suggested the step-by-step approach for the enhancement of awareness about VVAPs in medical education. It would be better to incorporate VVAPs in the medical curriculum and postgraduate training, since many courses are spreading medical knowledge of marketing and conflict of interests. In medical schools, there is a need to integrate the course about aesthetics surgery, especially at the clerkship phase, as shown in Fig. 3 .

Furthermore, during the clinical rotations for medical students, there must be an option for rotation to aesthetic gynecology clinics. During postgraduate training, there should be the choice of attachments at VVA clinics for gynecology trainees. Besides, the arrangements of multidisciplinary meetings (gynecology, cosmetology, plastic surgery) can help the postgraduate trainee to develop the skills of decision making about different procedures based on the signs and symptoms of the patients. The arrangements of workshops and conferences on VVAPs can enhance knowledge and provide opportunities for health professionals to exchange their views. Clinical training in the VVAPs subspecialty in the form of VVAPs courses, medical ethics VVAPs courses, and regular awareness campaigns can enhance education among health professionals.

## Challenges

Currently, the biggest challenge for health professionals is the business model approach to VVAPs. The business of pursuing beauty for a perfect body can go further than the body-improving products and practices. For some women, modifying their bodies has become normalized, and “designer vagina” has become a public word, increasing the trend for female genital cosmetic surgery. 25 Mostly, women are being misled about the normal appearance of genitalia and its normal variations, desiring a pre-pubertal, doll-like look, with nonapparent labia minora (not having it projected beyond the labia majora), no excess skin on the clitoris head, not much fat over the mons pubis, etc. This misleading is eventually showing the lack of awareness of treating physicians and healthcare providers rather than of patients. Being medical educators, we must opt for an evidence-based approach to create awareness among health providers and the public rather than support the business model. Further research is required in order to determine the sexual, psychological, and body image outcomes for women who underwent elective VVAPs. It is imperative to determine the benefits and impacts on sexual satisfaction, and the need for assessment of functional disorders before surgery. There is a requirement to conduct a qualitative and in-depth analysis to inquire about the reasons for ethical and medical justifications based on personal and cultural beliefs regarding VVAPs.

## Conclusion

Vulvovaginal aesthetics procedures have gained significant popularity among health professionals, different media platforms, and patients. In the reproductive age group, this topic has appeared as a fascinating area for discussion to increase self-esteem, and to rejuvenate genitals for the improvement of sexual function. The present study aimed to explore the awareness of VVAPs among health professionals and medical students in Saudi Arabia. There is a significant demand to educate doctors, health professionals, medical students, and gynecologists for the VVAPs to have a solid foundation, justified indications, and knowledge about various aesthetic options. Skilled health professionals should have optimal expertise to evaluate the clinical picture of the patients and involve multidisciplinary health teams for management. Although female doctors, medical students, young doctors, and gynecologists have more knowledge about VVAPs, all health professionals ought to be aware of recent trends in VVA. This analysis determined that VVA should be under the domain of gynecologists domain rather than under those of plastic surgeons, general surgeons, and cosmetologists. Most of the participants considered that vaginal rejuvenation, “G-spot” augmentation, clitoral surgery, and hymenoplasty are not justifiable on medical grounds. Regarding ethical considerations, more than half of the participants considered that vaginal rejuvenation, “G-spot” augmentation, clitoral surgery, and hymenoplasty are not ethically accepted. Additionally, the decision to opt for different techniques for vaginal tightening and revitalization should be taken very carefully, utilizing the shared decision-making approach. Ethical aspects and moral considerations are important key factors before embarking in VVAPs purely for cosmetic reasons. Further research is required to determine the sexual, psychological, and body image outcomes for women who underwent elective VVAPs. It is imperative to determine the benefits and impacts on sexual satisfaction postoperatively. Medical educators must consider VVAPs as part of the undergraduate and postgraduate curriculum in order to enhance awareness among medical professionals.
